# Synergistic Anticancer Effect of Tocotrienol Combined with Chemotherapeutic Agents or Dietary Components: A Review

**DOI:** 10.3390/ijms17101605

**Published:** 2016-09-22

**Authors:** Takahiro Eitsuka, Naoto Tatewaki, Hiroshi Nishida, Kiyotaka Nakagawa, Teruo Miyazawa

**Affiliations:** 1 Faculty of Applied Life Sciences, Niigata University of Pharmacy and Applied Life Sciences, Niigata 956-8603, Japan; tatewaki@nupals.ac.jp (N.T.); hnishida@nupals.ac.jp (H.N.); 2 Food & Biodynamic Chemistry Laboratory, Graduate School of Agricultural Science, Tohoku University, Sendai 981-8555, Japan; nkgw@m.tohoku.ac.jp; 3 Food and Biotechnology Innovation Project, New Industry Creation Hatchery Center (NICHe), Tohoku University, Sendai, Miyagi 980-8579, Japan; miyazawa@m.tohoku.ac.jp; 4 Food and Health Science Research Unit, Graduate School of Agricultural Science, Tohoku University, Sendai, Miyagi 981-8555, Japan

**Keywords:** vitamin E, tocotrienol, synergy, cancer

## Abstract

Tocotrienol (T3), unsaturated vitamin E, is gaining a lot of attention owing to its potent anticancer effect, since its efficacy is much greater than that of tocopherol (Toc). Various factors are known to be involved in such antitumor action, including cell cycle arrest, apoptosis induction, antiangiogenesis, anti-metastasis, nuclear factor-κB suppression, and telomerase inhibition. Owing to a difference in the affinity of T3 and Toc for the α-tocopherol transfer protein, the bioavailability of orally ingested T3 is lower than that of Toc. Furthermore, cellular uptake of T3 is interrupted by coadministration of α-Toc in vitro and in vivo. Based on this, several studies are in progress to screen for molecules that can synergize with T3 in order to augment its potency. Combinations of T3 with chemotherapeutic drugs (e.g., statins, celecoxib, and gefitinib) or dietary components (e.g., polyphenols, sesamin, and ferulic acid) exhibit synergistic actions on cancer cell growth and signaling pathways. In this review, we summarize the current status of synergistic effects of T3 and an array of agents on cancer cells, and discuss their molecular mechanisms of action. These combination strategies would encourage further investigation and application in cancer prevention and therapy.

## 1. Introduction

**Figure 1:**
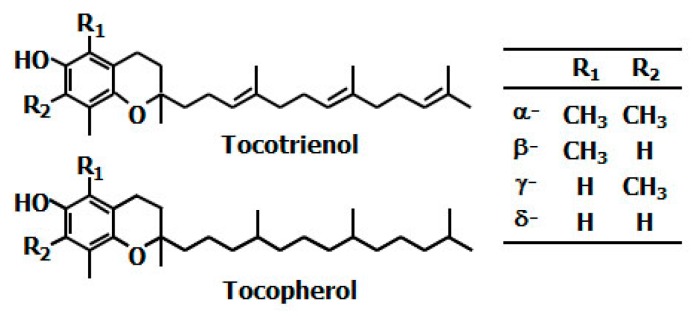
Chemical structure of vitamin E.

Vitamin E is divided into two groups, tocopherol (Toc) and tocotrienol (T3), both of which are made up of a chromanol ring with an isoprenoid-derived hydrophobic tail (Figure 1). Toc has a fully saturated phytyl tail, while T3 contains an unsaturated isoprenoid side chain with three double bonds. Both Toc and T3 occur naturally in four different forms: α-, β-, γ-, and δ-isomers, which are distinguished by the numbers and positions of a methyl group on the chromanol ring [1]. Toc is abundant in various foods including nuts, whole grains, green leafy vegetables, and common vegetable oils (e.g., olive, safflower, and sunflower oils). In contrast, T3 is present in a small fraction of plants; however, annatto, palm, and rice bran oils are known to be some of the richest sources of T3 [2].

T3 was first discovered and isolated from the latex of the rubber tree (*Hevea brasiliensis*) in 1964 [3]. Although the beneficial health effects of T3 were not evident over the following two decades, Qureshi et al. demonstrated for the first time that T3 possesses a cholesterol-lowering effect in 1986 [4]. Moreover, anti-carcinogenic and anti-proliferative actions of T3 were revealed in 1989 and 1995, respectively [5,6]. Since then, a number of researchers have investigated the mechanism of action of T3 against a variety of diseases, to clarify the broad beneficial activities of T3, such as its anti-oxidative [7], anti-tumor [6], anti-diabetic [8], anti-inflammatory [9], immune-stimulatory [10], cardio-protective [11], bone-protective [12], neuro-protective [13], hepato-protective [14], and nephro-protective effects [15]. These bioactivities of T3 are generally superior to those of Toc, since T3 is more efficiently incorporated into the lipid bilayer of the cell membrane compared with Toc, owing to its unsaturated isoprenoid chain [16].

Toc and T3 are absorbed in the small intestine, packaged into chylomicrons, and then secreted into the lymph and blood [17]. In the bloodstream, chylomicron triacylglycerol is hydrolyzed by lipoprotein lipase, forming chylomicron remnants. These are mainly taken up by the liver where α-tocopherol transfer protein (α-TTP) transfers vitamin E to very-low-density lipoproteins. Toc and T3 are then secreted into the blood again, and transported to various tissues. Although α-TTP exhibits the highest affinity for α-Toc among all vitamin E isomers, its binding affinity to α-T3 is 12% of that to α-Toc [18]. Thus, the bioavailability of orally administered T3 is lower than that of Toc. In fact, plasma concentrations of Toc and T3 are shown to reach 11–37 μM and 1 μM, respectively [19]. Moreover, cellular uptake of T3 is interrupted by coadministration of α-Toc in vitro [20] and in vivo [21], indicating that α-Toc decreases the bioavailability of T3.

Considering this, several studies are in progress to screen for molecules that can synergize with T3 in order to augment its potency. Combinations of T3 and certain drugs, such as statins [22], erlotinib/gefitinib [23], celecoxib [24], SU11274 [25], GW966/T0070907 [26], oridonin [27], and baicalein [28], have synergistic actions on cancer cell growth and signaling pathways. In addition, co-treatment with T3 and dietary components, including epigallocatechin gallate (EGCG)/resveratrol [29], sesamin [30], and ferulic acid [31], also exhibit synergistic effects. In this review, we present an overview of the agents that can potentiate the anticancer effects of T3 and their synergistic mechanisms of action.

## 2. Synergistic Anticancer Actions of T3 and Chemotherapeutic Drugs

### 2.1. Statins

**Figure 2:**
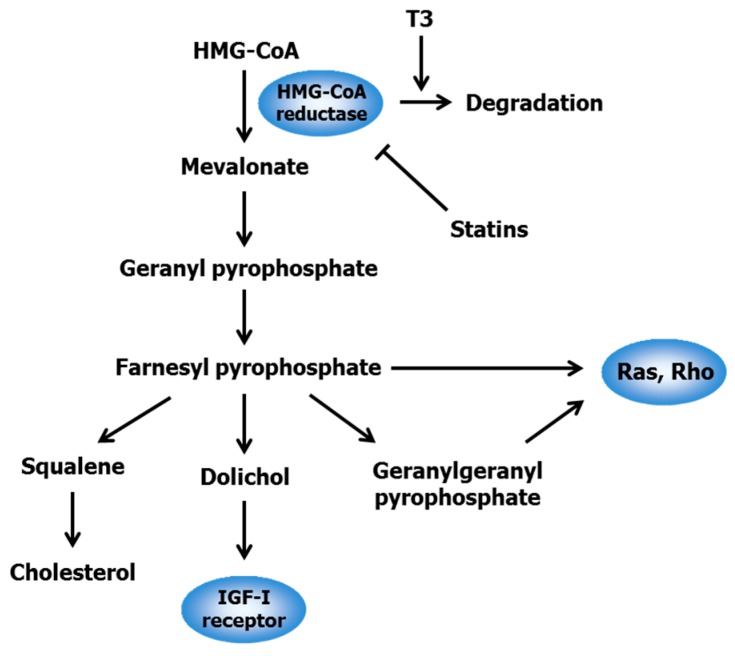
Mevalonate pathway and intermediates necessary for the posttranslational modification of Ras, Rho, and insulin-like growth factor I (IGF-I) receptor. T3, tocotrienol; HMG-CoA, 3-hydroxy-3-methylglutaryl-coenzyme A. Arrows and perpendicular lines indicate activation/induction and inhibition/suppression, respectively.

Statins, a class of drugs including lovastatin, simvastatin, mevastatin, and atorvastatin, can lower high blood cholesterol levels through competitive inhibition of 3-hydroxy-3-methylglutaryl-coenzyme A (HMG-CoA) reductase, the rate-limiting enzyme of the mevalonate pathway for cholesterol synthesis [32]. This pathway provides various isoprenoid intermediates including farnesyl pyrophosphate, geranylgeranyl pyrophosphate, and dolichol, all of which play an important role in cell survival and growth (Figure 2). Farnesylation and geranylgeranylation of Ras- and Rho-family proteins lead to their membrane anchorage, which is essential to their activation and initiation of downstream signaling pathways [33]. Ras proteins participate in regulating cell proliferation and survival, while Rho proteins are involved in the control of cell motility and cell-cell adhesion. Dolichol is responsible for the N-linked glycosylation and translocation of insulin-like growth factor I (IGF-I) receptors to the cell surface, thereby leading to cell proliferation [34]. Hence, the mevalonate pathway contributes to posttranslational modification and maturation among Ras, Rho, and IGF-I receptor proteins that regulate cell cycles, apoptosis, and metastasis. Intriguingly, HMG-CoA activity is dysregulated and up-regulated in various tumor tissues [35]. Statins can, therefore, suppress the proliferation of several types of cancer cells by inducing G1 arrest and/or apoptosis [35], indicating that statins exhibit not only cholesterol-lowering but also anticancer effects.

T3, particularly the γ- and δ-isomers, has been shown to attenuate cholesterol synthesis through posttranscriptional down-regulation of HMG-CoA reductase [36,37]. Although δ-T3 dose-dependently suppressed the proliferation of MIA PaCa-2, BxPC-3, and PANC-1 cells, supplementation with mevalonate, the product of HMG-CoA reductase, diminished the δ-T3-mediated growth inhibition of these cells [38]. Given that statins and T3 attenuate HMG-CoA reductase activity via a different mechanism of action, co-treatment with these agents is thought to exert additive or synergistic inhibitory effects on tumor growth. Mo and Elson [22] discovered for the first time that co-treatment with γ-T3 and lovastatin resulted in a synergistic decrease in the growth of DU145 and LNCaP human prostate cancer cells. McAnally et al. [39] reported that combined treatment with T3 and lovastatin synergistically suppressed the proliferation of B16 (murine melanoma), DU145 (human prostate carcinoma), and A549 (human lung carcinoma) cells. Furthermore, coadministration of T3 and lovastatin synergistically suppressed the growth of B16 cells implanted in C57BL6 mice. Wali and Sylvester [40] revealed that a single treatment with 3–4 μM γ-T3 or 2–8 μM statins (i.e., simvastatin, lovastatin, or mevastatin) significantly decreased the growth of neoplastic +SA mouse mammary epithelial cells, whereas for combinations of 0.25–2 μM γ-T3 and 0.25 μM each statin exhibited a synergistic inhibitory effect via suppression of mitogen-activated protein kinase (MAPK), c-Jun N-terminal kinase JNK, p38, and protein kinase B (Akt). Moreover, the growth-inhibitory effect of γ-T3 on HT29 and HCT116 colon cancer cells was enhanced by atorvastatin through cell cycle arrest at G1 phase and apoptosis [41], and co-treatment with δ-T3 and lovastatin caused a synergistic decrease in the growth of A2058 melanoma cells [42].

### 2.2. Gefitinib and Erlotinib

**Figure 3:**
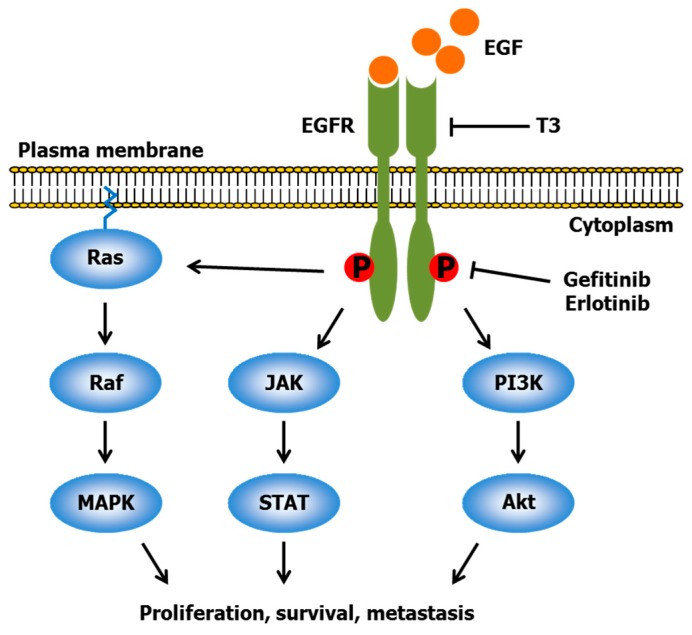
Epidermal growth factor receptor (EGFR) and its downstream signaling proteins. EGF, epidermal growth factor; EGFR, epidermal growth factor receptor; JAK, Janus kinase; STAT, signal transducer and activator of transcription; PI3K, phosphatidylinositol 3-kinase; MAPK, mitogen-activated protein kinase; Akt, protein kinase B; P, phosphorylation. Arrows and perpendicular lines indicate activation/induction and inhibition/suppression, respectively.

Gefitinib and erlotinib are well-known epidermal growth factor receptor (EGFR) tyrosine kinase inhibitors [43]. EGFR, a plasma membrane glycoprotein, is a member of the ErbB receptor family, and is composed of an extracellular ligand-binding domain, a lipophilic transmembrane domain, and an intracellular cytoplasmic domain with tyrosine kinase [44]. EGFR is activated by some ligands including epidermal growth factor (EGF), transforming growth factor-α, heparin-binding EGF, and amphiregulin. Ligand binding to the extracellular domain of EGFR induces a conformational change in the intracellular cytoplasmic domain, which promotes homodimerization or heterodimerization with the other ErbB family members, leading to the autophosphorylation of tyrosine residues and phosphorylation and the activation of downstream signaling pathways (Figure 3) [45]. These pathways include Ras/Raf/MAPK, phospholipase C/phosphatidylinositol 3-kinase (PI3K)/Akt, and Janus kinase/signal transducer and activator of transcription (JAK/STAT) [46]. The MAPK pathway is implicated in cell proliferation and survival. The PI3K/Akt pathway is involved in cell proliferation and migration. The JAK/STAT pathway participates in the transcription of genes involved in oncogenesis. Abnormal activation and overexpression of EGFR occur in various types of tumor cells, resulting in the enhancement of cell growth, survival and metastasis [47]. Gefitinib and erlotinib can competitively inhibit the binding of adenosine triphosphate to EGFR kinase, which causes the inhibition of autophosphorylation and downstream pathways [43]. Therefore, both drugs exert potent antitumor activity, and have been approved for first-line treatment in patients with lung adenocarcinoma with mutated EGFR.

T3 down-regulates the ErbB family receptors and downstream pathways. Samant and Sylvester [48] showed that γ-T3 dose-dependently inhibited +SA cell growth, and revealed that its inhibitory effect was involved in the suppression of the PI3K/Akt pathway through a significant decrease in ErbB3 receptor tyrosine phosphorylation. T3, but not Toc, attenuated PI3K/Akt and MAPK pathways via downregulation of ErbB2 expression, thereby leading to the induction of apoptosis in pancreatic cancer cells [49]. Pierpaoli et al. [50] examined the effect of dietary supplementation with T3 extracts from annatto seeds (10% γ- and 90% δ-T3 mixture) on the spontaneous development of mammary tumors in ErbB2 transgenic mice. Oral administration of annatto-T3 diminished the size of mammary tumors, and induced apoptosis and senescence-like growth arrest of tumor cells. In a cell culture experiment, the suppression of breast cancer cell growth, increased apoptosis and senescence molecular markers (p53, p21, p16, and p27), and a decreased expression of ErbB2 was observed in the cells treated with annatto-T3. Alawin et al. [51] revealed that γ-T3 interfered with the dimerization and phosphorylation of ErbB2 via its accumulation in the lipid raft microdomain, resulting in the inhibition of the proliferation in SKBR3 and BT474 human breast cancer cells.

There are limited reports that have investigated whether EGFR inhibitors (i.e., gefitinib and erlotinib) can synergize with T3. Treatment with 3.5 μM γ-T3, 0.5 μM erlotinib or 1.0 μM gefitinib significantly repressed the proliferation of +SA cells. The combination of 0.5–3 μM γ-T3 with 0.5 μM erlotinib or 1.0 μM gefitinib synergistically inhibited cell growth and elicited apoptosis through the activation of caspase-3. These synergistic inhibitory effects were mediated by a marked reduction in ErbB2-4 levels [23]. Bachawal et al. [52] also clarified that co-treatment with γ-T3 and erlotinib/gefitinib resulted in a synergistic decrease in +SA cell growth through down-regulation of ErbB receptors and downstream signaling of Akt and STAT.

### 2.3. Celecoxib

**Figure 4:**
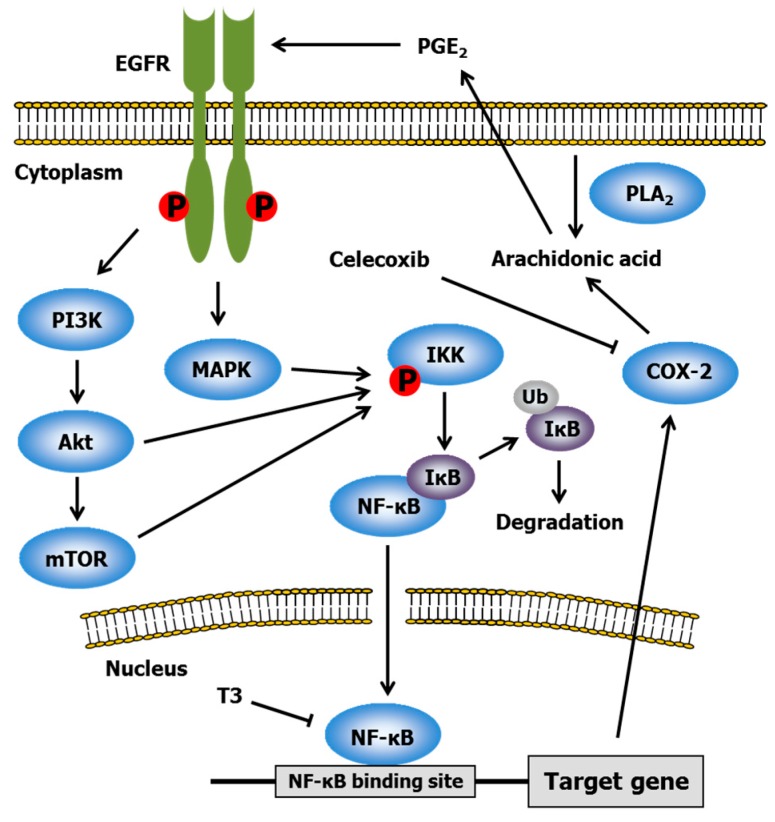
Crosstalk between EGFR and nuclear factor-κB (NF-κB) signaling pathway. PGE_2_, prostaglandin E_2_; IκB, inhibitors of κB; IKK, IκB kinase; COX-2, cyclooxygenase-2; mTOR, mammalian target of rapamycin; Ub, ubiquitylation; PLA_2_, phosphorlipase A_2_; P, phosphorylation. Arrows and perpendicular lines indicate activation/induction and inhibition/suppression, respectively.

Celecoxib, a non-steroidal anti-inflammatory drug, is a specific inhibitor of cyclooxygenase-2 (COX-2) [53]. Aberrant activation of COX-2 is observed in gastric, hepatocellular, esophageal, pancreatic, colorectal, breast, bladder, cervical, endometrial, skin, and lung cancers, and is involved in promoting cell survival, angiogenesis, and metastasis [54]. Therefore, the elucidation of downstream signaling of COX-2 is important for understanding cancer progression. COX-2 metabolizes arachidonic acid to prostaglandin H_2_, which can be isomerized to prostaglandin E_2_ (PGE_2_) by PGE synthase. Interestingly, PGE_2_ activates EGFR via a Src-dependent mechanism [55]. This finding indicates that COX-2 inhibitors have cancer-fighting activity, since EGFR is recognized as a target for cancer therapy as described above. It is well established that EGFR phosphorylation induces nuclear factor-κB (NF-κB) activation (Figure 4) [56]. NF-κB is one of the major pro-inflammatory transcriptional factors. In unstimulated cells, inhibitors of κB (IκB) bind to NF-κB, resulting in its cytosolic location in an inactive form. In response to cell stimulation, IκB proteins are phosphorylated by IκB kinase (IKK), leading to IκB ubiquitination and degradation, thereby allowing active NF-κB to translocate to the nucleus. Various kinases, such as MAPK, Akt and mammalian target of rapamycin (mTOR), can phosphorylate IKK, which eventually causes NF-κB activation [57]. NF-κB target genes participate in the regulation of inflammation (e.g., COX-2, tumor necrosis factor-α, and interleukin-1), cell proliferation (e.g., Cyclin D1, Cyclin E, and c-Myc), antiapoptosis (e.g., Bcl-xL, inhibitor of apoptosis protein, and tumor necrosis factor receptor-associated factor), metastasis (e.g., matrix metalloproteinase and urokinase plasminogen activator), and angiogenesis (e.g., interleukin-1 and vascular endothelial growth factor) [58]. The findings noted above indicate that celecoxib can inhibit NF-κB as well as COX-2. In addition, celecoxib prevents NF-κB activation via the inhibition of IKK and Akt, resulting in the repression of COX-2 synthesis and various genes required for inflammation and proliferation [59].

The effects of T3 on NF-κB have been extensively studied in several types of tumor cells such as mammary epithelial [60], myeloma [61], prostate [62], colon [63], melanoma [64], breast [65], pancreatic [66], lung [67], gastric [68], and oral cancer cells [69]. Ahn et al. [61] found that γ-T3 but not γ-Toc decreased NF-κB activation through the repression of the phosphorylation and degradation of IκB, inhibition of IKK activation by blocking activation of transforming growth factor-β-activated kinase 1, and attenuation of the phosphorylation and nuclear translocation of p65, an NF-κB family member. Its inhibitory action was correlated with the down-regulation of NF-κB target gene expression related to proliferation, antiapoptosis, invasion, and angiogenesis. Ji et al. [67] showed that treatment of lung cancer cells with δ-T3 caused a reduction in NF-κB-DNA binding activity and down-regulation of NF-κB-dependent gene expression including genes coding for surviving, matrix metalloproteinase-9, vascular endothelial growth factor, and Bcl-xL. Wang et al. [70] revealed that γ-T3 inhibited NF-κB activation via induction of A20 and/or Cezanne, both of which act as strong inhibitors of NF-κB activity.

A synergistic effect between T3 and celecoxib was discovered by Shirode and Sylvester. Although 3–4 μM γ-T3 or 7.5–10 μM celecoxib alone significantly inhibited +SA cell growth in a dose-dependent manner, co-treatment with 0.25 μM γ-T3 and 2.5 μM celecoxib had synergistic action [24]. The anti-proliferative effect was mediated by a decrease in PGE_2_ synthesis and reduced levels of COX-2, Akt, and NF-κB. They also clarified that the synergy between γ-T3 and celecoxib was due to the suppression of ErbB2-4 receptor levels and following reduction of downstream Akt and NF-κB signaling [71].

### 2.4. Other Drugs

Mesenchymal epithelial transition factor (Met), also known as hepatocyte growth factor (HGF) receptor, is the cell surface receptor for HGF, which possesses tyrosine kinase activity [72]. The binding of HGF induces homodimerization of the Met receptor and phosphorylation of a tyrosine residue within the catalytic site, leading to the activation of downstream signaling pathways, including MAPK, Akt, and STAT [73]. Met is known to interact with EGFR, thereby inducing a diverse series of signaling cascades [74]. Several types of tumor cells exhibit sustained Met stimulation, overexpression, or mutation [73]. Hence, Met plays a key role in cancer cell growth and survival. Ayoub et al. revealed that γ-T3 repressed HGF-induced Met tyrosine kinase activation and signaling in breast cancer cells (+SA, MCF-7, and MDA-MB-231) [25,75]. A combination of γ-T3 and SU11274 (a Met inhibitor) synergistically suppressed cell proliferation through the reduction in Akt, STAT, and NF-κB activation.

The peroxisome proliferator-activated receptor γ (PPARγ) belongs to the nuclear hormone receptor superfamily, which acts as a transcription factor after it heterodimerizes with the retinoid X receptor [76]. PPARγ can be activated by ligands such as 15-deoxy-Δ^12,14^-prostaglandin J_2_ and some unsaturated fatty acids [77]. The receptor is present in various tissues and cell types throughout the body, including monocytes, macrophages, adipocytes, liver, skeletal muscle, breast, prostate, colon, as well as cancer cells [77]. Most of the target genes of PPARγ participate in the control of lipid metabolism and transport. In addition, PPARγ can attenuate the expression of pro-inflammatory transcription factors such as NF-κB and activator protein-1 (AP-1) [78]. Synthetic PPARγ ligands inhibit an array of cancer cells in vitro and in vivo [77], suggesting an important role of PPARγ as a tumor suppressor; however, the precise mechanism is still unclear. Campbell et al. reported that T3, especially γ- and δ-isomers, repressed the proliferation of prostate cancer cells (PC-3 and LNCaP) more effectively compared with Toc, while PPARγ knockdown diminished the anti-proliferative effect of T3 [79]. T3 induced 15-lipoxygenase-2 that is responsible for the production of 15-S-hydroxyeicosatrienoic acid, a PPARγ-activating ligand. These results suggested that T3 inhibited prostate cancer cell growth through, in part, PPARγ-dependent mechanisms. Malaviya and Sylvester examined the anti-proliferative effect of γ-T3 in combination with PPARγ agonists or antagonists on breast cancer cells (MCF-7 and MDA-MB-231) [26]. Single treatment with γ-T3, PPARγ agonists (rosiglitazone or troglitazone) or antagonists (GW9662 or T0070907) resulted in a dose-dependent growth inhibition of breast cancer cells. Unexpectedly, a combination with γ-T3 and the agonists promoted cancer cell proliferation, whereas co-treatment of γ-T3 and the antagonists synergistically suppressed the cell growth. The contradictory results might be derived from the cancer cell types used in this experiment; the authors [26] employed breast cancer cells while Campbell et al. [79] used prostate cancer cells. They concluded that the synergistic effect between γ-T3 and PPARγ antagonists was mediated through PPARγ-independent mechanisms [80].

Autophagy is a cellular self-catabolic process by which dysfunctional or unnecessary cytoplasmic components are degraded by lysosomal enzymes [81]. The process is initiated by the formation of the isolation membrane (also known as the phagophore) to engulf damaged protein aggregates and intracellular organelles. The isolation membrane expands and closes to form a double-membrane vesicle (i.e., autophagosome), leading to the fusion of autophagosomes with lysosomes. Finally, the inner contents are degraded and recycled. Although autophagy maintains intracellular homeostasis, excessive autophagy disturbs normal cellular function and induces cell death [82]. The autophagic pathway includes mTOR, class I PI3K, Akt, class III PI3K, Beclin-1, Atg family member proteins, and p53 [83]. γ-T3 has been shown to induce apoptosis and autophagy in prostate and breast cancer cells [84,85]. Tiwari et al. investigated the synergistic action of γ-T3 and oridonin, an autophagy inducer [86], against +SA mammary cancer cells. Co-treatment with γ-T3 and oridonin synergistically decreased cell viability via the elevation of autophagy markers (e.g., Beclin-1 and Atg), suppression of Akt/mTOR signaling, and up-regulation of apoptotic markers (e.g., caspase-3 and Bax/Bcl-2 ratio) [27].

The aryl hydrocarbon receptor (AhR) is a ligand-activated transcription factor, which regulates cell differentiation, proliferation, immune response, and epidermal barrier function [87]. Ligand-bound AhR complexes translocate to the cell nucleus to interact with AhR nuclear translocator (ARNT), forming a functional AhR-ARNT heterodimer, resulting in the transcriptional activation of several target genes (e.g., p21 [88] and Bax [89]) by binding to the xenobiotic responsive element. In B16F10 mouse melanoma cells, stable knockdown of AhR enhanced tumorigenesis and metastatic potential to the lungs, while constitutive AhR activation potently inhibited melanoma progression [90]. Baicalein, one of flavones, is a component of the traditional herbal remedy known as Chinese skullcap (*Scutellaria baicalensis*). Baicalein can act as a ligand of AhR, and exhibits anticancer action through, in part, AhR activation [91]. Yamashita et al. found that γ-T3 dose-dependently induced the expression of AhR in B16 mouse melanoma cells [28]. Thus, combination treatment with γ-T3 and baicalein synergistically inhibited the cell growth via induction of p21 and Bax expressions.

## 3. Synergistic Anti-Proliferative Effects of T3 and Dietary Components

### 3.1. Epigallocatechin Gallate (EGCG) and Resveratrol

EGCG and resveratrol are well-known dietary polyphenols. EGCG is present in tea, grapes, and certain seeds of leguminous plants [92], while resveratrol is naturally found in grapes, peanuts, berries, and red wine [93]. Both polyphenols have diverse health-promoting effects, particularly anticancer action [94,95], similar to T3. The molecular mechanisms of the cancer-fighting properties of these three dietary compounds overlap each other. For example, EGFR is one of the critical targets of EGCG in the repression of cancer proliferation [96]. EGCG attenuates the activation of ErbB family receptors via the inhibition of tyrosine kinase activity and EGF binding to the EGFR, leading to a down-regulation of the MAPK and Akt signaling pathways. EGCG also down-regulates NF-κB and AP-1, thereby modulating the expression of target genes involved in apoptosis and cell cycle regulation. Similarly, resveratrol can suppress EGFR activation [97]. In addition, resveratrol down-regulates the mRNA expression of HMG-CoA reductase [98], and combined treatment with resveratrol and simvastatin synergistically reduces cell proliferation [99].

Hsieh and Mu [29] tested the synergistic anti-proliferative effect of EGCG, resveratrol, and γ-T3 on breast cancer cells. Treatment with resveratrol or γ-T3 alone (10 μM each) significantly inhibited cell growth, but treatment with EGCG did not show any effect. Resveratrol potentiated γ-T3-induced inhibition of cell proliferation. However, effective suppression of the cell growth was not observed when the three phytochemicals were concomitantly used. Co-treatment with γ-T3 with either EGCG or resveratrol caused a significant additive effect in reducing cyclin D1 and Bcl-2 expression. The triple combination of EGCG, resveratrol and γ-T3 synergistically induced NAD(P)H quinone dehydrogenase 1 (NQO1). NQO1 is involved in phase II detoxification, and its expression is regulated by nuclear factor erythroid 2–related factor 2 (Nrf2) [100]. Nrf2, a transcriptional factor, is maintained in an inactive form in the cytoplasm by binding to Kelch-like ECH-associated protein 1 (Keap1). During redox imbalance, Nrf2 dissociates from Keap1, resulting in the translocation of Nrf2 to the nucleus and heterodimerization with Maf to bind to antioxidant response elements in various promoter regions, which increases the transcription of a variety of cytoprotective genes (e.g., glutathione S-transferases, heme oxygenase-1, and NQO1) [101]. Hence, Nrf2 acts as a central regulator of the adaptive response to oxidative stress. EGCG, resveratrol, and γ-T3 can individually up-regulate Nrf2 [102,103,104]. However, it is unclear how the three phytochemicals synergistically increase NQO1 expression [29].

### 3.2. Sesamin

Sesamin, one of the major lignans in sesame seed and flax, possesses various beneficial functions, including anti-oxidative [105], lipid-lowering (arachidonic acid [106] and cholesterol [107] levels), anti-hypertensive [108], neuroprotective [109], anti-tumor [110], and anti-inflammatory [111] actions. Sesamin also plays a unique role in improving the bioavailability of T3 in vitro [112] and in vivo [113].

**Figure 5:**
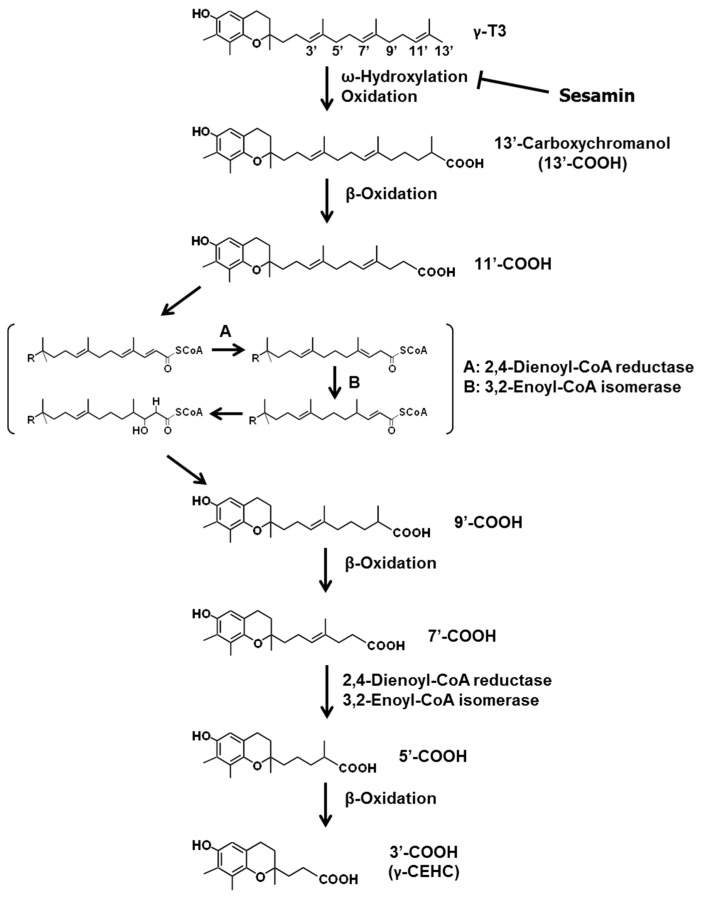
Metabolic pathway of γ-T3. The second and fourth cycles of β-oxidation are needed for 2,4-dienoyl-CoA reductase (A) and 3,2-enoyl-CoA isomerase (B). Arrows and perpendicular lines indicate activation/induction and inhibition/suppression, respectively.

All vitamin E isoforms are catabolized through the oxidative degradation of their side chains to form water-soluble metabolites known as carboxyethyl hydroxychromans (CEHCs). The degradation begins with cytochrome P450 (CYP)-catalyzed ω-hydroxylation. In humans, CYP4F2 [114] has been reported to mediate this metabolic process, and CYP3A [115] might also be involved. These CYPs catalyze the hydroxylation of one of the terminal methyl groups on the hydrophobic side chain. The ω-hydroxylated Toc and T3 are then oxidized to the corresponding carboxylic acid, followed by five cycles of β-oxidation to eliminate a two-carbon moiety from the side chain at each cycle, ultimately leading to the formation of CEHCs (Figure 5) [116,117]. Most CEHCs are excreted in the urine [17]. Some CYP inhibitors can markedly elevate the levels of vitamin E and lower the levels of vitamin E metabolites in various tissues. Ketoconazole is a common inhibitor of CYP3A and 4F. Coadministration of ketoconazole and vitamin E (Toc and T3) decreased urinary excretion of CEHC and increased Toc and T3 concentrations in the serum and various tissues of rats [118]. In addition, sesamin strongly inhibits CYP4F2 activity, thereby elevating vitamin E concentrations in rat and human liver microsomes [114]. Thus, the bioavailability of Toc and T3 is improved by the addition of sesamin.

Akl et al. [30] investigated the synergistic inhibition of +SA mammary cancer cell proliferation with a combination of γ-T3 and sesamin. Treatment with 3.5–5 μM γ-T3 or 60–120 μM sesamin alone caused a significant inhibition of the cell growth. Combined treatment with 1–5 μM γ-T3 and 20 μM sesamin resulted in a synergistic suppression of +SA proliferation, indicating that an increase in T3 bioavailability by sesamin caused an enhancement of its anticancer activity. Co-treatment of γ-T3 and sesamin markedly prevented the activation of the ErbB receptor and its downstream signaling molecules (i.e., Raf, PI3K, Akt, NF-κB, JAK, and STAT), indicating that the synergistic action was associated with the EGF-dependent pathway. Akl et al. [119] also revealed that the combination of γ-T3 and sesamin synergistically repressed the proliferation of breast cancer cells (+SA, MCF-7, and MDA-MB-231) via the induction of G1 cell cycle arrest; however, this had no effects on normal epithelial cell growth. The combined treatment efficiently influenced cell cycle regulators of the G1/S phase transition; cyclin D1, cyclin-dependent kinase (CDK) 2, CDK4, CDK6, phospho-retinoblastoma, and E2F1 levels were reduced, and p27 and p16 levels were increased.

### 3.3. Ferulic Acid

The bran fraction of rice contains a variety of bioactive components with chemopreventive activity, including T3, ferulic acid, γ-oryzanol, β-sitosterol, and squalene [120]. Numerous studies have been performed to investigate the anticancer properties of dietary rice bran. Tantamango et al. [121] reported that the consumption of certain foods was associated with the decreased risk of development of polyps in a prospective study. In addition to the protective effect of green vegetables, dried fruit, and legumes, this study found that consumption of brown rice had the strongest correlation with a reduced risk of polyp formation.

We showed that T3 can act as an effective anti-tumor compound (i.e., antiangiogenesis [122] and telomerase inhibition [123]), and demonstrated that δ-T3 exhibits the most potent anti-cancer property among the four T3 isomers. Ferulic acid is receiving attention owing to its wide range of therapeutic effects against cancer [124], diabetes [125], cardiovascular diseases [126], and neurodegenerative disorders [127]. Although rice bran has a broad spectrum of beneficial activity for human health, little is known about the synergistic effect of rice bran components on cancer cell proliferation to date. We, therefore, investigated the potential role of its components, particularly δ-T3 and ferulic acid, in synergistic growth-inhibitory activity against an array of cancer cells, such as DU-145 human prostate carcinoma, MCF-7 human breast adenocarcinoma, and PANC-1 human pancreatic carcinoma cells [31].

δ-T3 dose-dependently inhibited the proliferation of these three cell lines, while ferulic acid exhibited no growth inhibition even at a concentration of 50 μM. Combined treatment with 10–12.5 μM δ-T3 and 5–20 μM ferulic acid markedly reduced cell growth compared with treatment with δ-T3 alone, although 20 μM ferulic acid had no inhibitory effect at all. Ferulic acid enhanced δ-T3-induced G1 phase arrest through the up-regulation of p21, a negative regulator of G1 progression.

To elucidate the reason for the synergistic inhibition of cell proliferation by co-treatment with δ-T3 and ferulic acid, the intracellular content of δ-T3 was analyzed by high performance liquid chromatography (HPLC). Co-treatment with δ-T3 and ferulic acid increased the cellular concentration of δ-T3 in PANC-1 cells compared to treatment with δ-T3 alone. The increment of cellular δ-T3 levels in PANC-1 implies that ferulic acid either suppresses the intracellular metabolism of δ-T3 or facilitates cellular uptake of δ-T3 from the cell culture medium. To address the latter possibility, we evaluated δ-T3 concentrations in a culture medium using HPLC, and found that ferulic acid treatment did not influence the amount of δ-T3 in the culture medium [31]. It seems, therefore, unlikely that ferulic acid affects cellular δ-T3 incorporation. These findings suggest that sesamin and ferulic acid may synergize with T3 through a similar mode of action (i.e., increasing the concentration of T3). Zhao et al. [128] investigated the bioavailability of ferulic acid in rats administered 70 μmol/kg of ferulic acid, and revealed that the plasma concentration of ferulic acid was 25.3 ± 10.1 μM at 5 min after administration. These observations indicate that physiological concentrations of ferulic acid can potentiate the growth-inhibitory effects of δ-T3 on several types of cancer cells, and suggest that ferulic acid may be a promising candidate for augmenting the anti-cancer activity of δ-T3.

Moreover, we hypothesized that combined treatment with δ-T3 and ferulic acid would not only inhibit cancer cell growth but would also enhance various physiological activities of δ-T3. We additionally investigated whether the combination of δ-T3 and ferulic acid synergistically down-regulated telomerase activity in DLD-1 colorectal cancer cells [129]. As expected, co-treatment with δ-T3 and ferulic acid resulted in a synergistic reduction of cellular telomerase activity via a decreased expression of human telomerase reverse transcriptase, the catalytic subunit of telomerase. Taken together, these results indicate that ferulic acid improves the bioavailability of T3, thereby synergistically suppressing cancer cell proliferation [31] and cellular telomerase activity [129].

## 4. Conclusions

Toc is widely present in various foods; however, T3-containing foods are limited. The daily intake of T3 in a Japanese population was estimated at 1.86–2.15 mg/day/person, which appeared relatively low compared with that of Toc (9 mg/day/person) [130]. Little is known about the influence of Toc on the anticancer effect of T3. In an attempt to clarify whether Toc affects the anti-proliferative activity of T3, DLD-1 cells were treated with both Toc isomers and δ-T3. All Toc isomers, especially α-Toc, diminished δ-T3-induced cytotoxicity to DLD-1 cells [20]. Co-treatment with α-Toc dose-dependently decreased δ-T3 uptake into the cells. These results indicate that α-Toc is not only less cytotoxic to cancer cells, but also reduces the cytotoxicity of δ-T3 by interfering with its cellular uptake. Findings of this in vitro study are supported by other in vivo work that found that α-T3 concentrations in various tissues and plasma decreased by the addition of dietary α-Toc, in the rats fed both α-T3 and α-Toc [21]. These findings raise concerns about the chemopreventive activity of T3 in vivo, since α-Toc is ubiquitously present as the dominant vitamin E isomer in animals. Hiura et al. [131] reported that dietary supplementation of pure T3 effectively suppressed tumor growth in a mouse xenograft model, suggesting that a high dose of T3 might overcome the inhibitory effects of endogenous α-Toc.

As we described above, chemotherapeutic drugs (i.e., HMG-CoA reductase inhibitors, EGFR tyrosine kinase inhibitors, a COX-2 inhibitor, a Met inhibitor, PPARγ antagonists, an autophagy inducer, and an AhR modulator) or dietary components (polyphenols, sesamin, and ferulic acid) potentiate the anticancer properties of T3. Combination therapy has an advantage in reducing the toxic adverse side effects of drugs associated with high-dose monotherapy. The clinical application of dietary compounds in cancer prevention is attractive, because they are non-toxic at physiological doses. Of note, a clinical trial conducted in humans resulted in no toxicities when up to 800 mg/day of δ-T3 was administered for several months [132]. Nesaretnam et al. [133] performed a pilot clinical trial to examine the effect of a T3-rich fraction (200 mg/day) and tamoxifen (20 mg/day) for 5 years in women with early breast cancer. Although the risk of dying due to breast cancer was lowered by 60% in patients treated with the combination of T3-rich fraction and tamoxifen compared to patients treated with placebo and tamoxifen, this was not statistically significant. Estrogen receptor (ER) status is a very important factor in planning breast cancer treatment. Two ERs, ERα and ERβ, are expressed in normal breast tissue, but the ratio of ERα to ERβ is elevated in breast tumors [134]. Therefore, selective ER modulators such as tamoxifen are currently used to treat breast cancer. In ERα-positive T47-D and MCF-7 human breast cancer cells, overexpression of ERβ not only attenuated Akt signaling via down-regulation of ErbB2/ErbB3 but also improved the sensitivity of these cancer cells to tamoxifen [135]. Comitato et al. [136,137] reported that T3-rich fraction from palm oil promoted ERβ translocation into nucleus, leading to the induction of apoptosis in MDA-MB-231 and MCF-7 breast cancer cells. These findings suggest that ER might be a promising target for breast cancer therapy by T3.

In this review, we summarized current research on the synergistic anti-tumor effect of T3 and various agents, and discussed their related mechanisms. Further studies, particularly animal and clinical tests, on the combination therapy of T3 and certain agents will contribute to their applications in cancer treatment and prevention.
